# Protocol for *in vitro* modeling of specification and morphogenesis of early pancreas development using human pluripotent stem cell-based organoid differentiation

**DOI:** 10.1016/j.xpro.2025.103834

**Published:** 2025-05-21

**Authors:** Chenglei Tian, Ulf Tiemann, Florian Hermann, Henrik Semb

**Affiliations:** 1 Institute of Translational Stem Cell Research, Helmholtz Diabetes Center, Helmholtz Zentrum Munchen, 85764 Munich, Germany; 2 Novo Nordisk Foundation Center for Stem Cell Biology (DanStem), University of Copenhagen, 2200 Copenhagen, Denmark

**Keywords:** Cell Biology, Cell culture, Developmental biology, Stem Cells, Cell Differentiation, Organoids

## Abstract

Here, we present a protocol to generate key pancreatic cell types *in vitro* using human pluripotent stem cell (hPSC)-based Matrigel-overlay organoid differentiation. These include multipotent and bipotent progenitors, endocrine progenitors, and hormone-producing endocrine cells. We describe steps for culturing hPSCs as a 2D monolayer, applying a Matrigel overlay to create a 3D epithelial niche, and guiding stepwise differentiation. This system supports live-cell imaging and real-time tracking of morphogenesis and fate decisions, providing a platform for studying organ development and disease.

For complete details on the use and execution of this protocol, please refer to Ulf et al.^1^

## Highlights

• Steps for culturing human pluripotent stem cells for differentiation
• Instructions for establishing Matrigel-overlay organoid differentiation system
• Instructions on differentiating human pluripotent stem cells into beta cells
• Guidance on characterization of differentiation efficiency and epithelial features

## Abstract (editor highlights)

Publisher’s note: Undertaking any experimental protocol requires adherence to local institutional guidelines for laboratory safety and ethics.

## Graphical abstract

**Figure:**
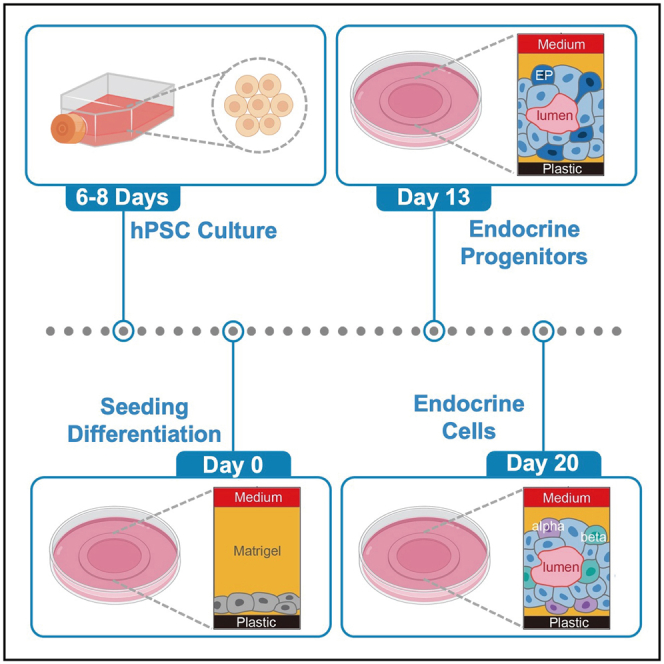

## Before you begin

Pancreatic development is driven by a complex interplay between intrinsic cellular properties and extrinsic cues that guide progenitor cells through differentiation.^2^^,^^3^^,^^4^^,^^5^ Conventional protocols for generating beta cells from human pluripotent stem cells (hPSCs) primarily focus on modulating intracellular signaling pathways using small molecules or cytokines in a stepwise manner.^6^^,^^7^^,^^8^ However, these approaches often overlook the critical role of extracellular signals, relying on empirical methods informed by pancreatic developmental biology and iterative optimization to achieve beta cell differentiation. Additionally, the lack of 3D architectural details in these protocols limits their ability to study how extracellular cues and morphological changes regulate early pancreatic development.

To address these limitations, we developed an hPSC-based Matrigel-overlay 3D organoid differentiation system that recapitulates *in vivo* epithelial luminal structures during endocrinogenesis. This system enables real-time live-cell imaging, allowing precise monitoring of dynamic cellular and morphological changes, as well as the interplay between intracellular and extracellular signaling pathways. By providing a physiologically relevant model for pancreas development, this approach enhances our understanding of fate decisions in progenitors, offering potential applications for refining differentiation protocols, disease modeling, and regenerative medicine strategies.

### Institutional permissions

The human embryonic stem cell (hESC) line (SA121, RRID: CVCL_B296) used in this study was obtained from the Takara Bio (Y00020) with all necessary approvals. NEUROG3-EGFP hPSC line generated from SA121.^9^ The use of hESC lines and the subsequent experiments have been approved in accordance with relevant regulatory standards.

### Preparation

#### Matrigel-coated plates, reagents, and medium preparation

**Timing: 4–5 h**

At least 1 day before thawing the hPSCs.

**CRITICAL:** Prepare all necessary reagents and materials before thawing the hPSCs and starting the differentiation, and check that all equipment is calibrated and set up for the experiments.

1. Aliquot Matrigel hESC-Qualified Matrix.
  ***Note:*** The Matrigel matrix is a solubilized basement membrane extract derived from mouse sarcoma. It is rich in extracellular matrix components and various growth factors that support hPSC growth. The hESC-Qualified Matrigel Matrix has been validated for the culture of hPSCs.
  a. Thaw one bottle of Matrigel hESC-Qualified Matrix (5 mL/vial) on ice at 4°C for at least 12 h.
  b. Pre-chill 1.5 mL Eppendorf (EP) tubes and 1 mL tips at −20°C for at least 30 min.
  c. In the cell culture hood, aliquot Matrigel hESC-Qualified Matrix with pre-chilled tips into the EP tubes based on the dilution factor and immediately store the aliquoted Matrigel hESC-Qualified Matrix at −80°C.
    ***Note:*** The dilution factor can be found on the Corning website using the catalog number. For example, the dilution factor of catalog number #354277 is 270 μL. Accordingly, 270 μL of Matrigel hESC-Qualified Matrix should be aliquoted per EP tube.
2. Preparation of Matrigel hESC-Qualified Matrix-coated flasks.
  a. In the cell culture hood, dilute one aliquoted Matrigel hESC-Qualified Matrix in 24 mL DMEM/F12 medium.
    ***Note:*** Keep both the aliquoted and diluted Matrigel matrix on ice during use to prevent solidification. Diluted Matrigel hESC-Qualified Matrix can be stored at 4°C for up to 2 weeks.
  b. Use this diluted Matrigel hESC-Qualified Matrix to coat T25 (2 mL/flask) or T75 flask (6 mL/flask).
    ***Note:*** Ensure that the entire bottom surface of the flask is evenly coated with diluted Matrigel.
  c. Incubate the Matrigel hESC-Qualified Matrix-coated flasks at 37°C in an incubator with 5% CO_2_ for at least 15 min.
  d. After incubation, the flasks can either be used immediately or kept at 4°C for up to one week by replacing the medium with an equal volume of PBS without calcium and magnesium (PBS^−/−^).
    ***Note:*** If kept the coated flask at 4°C in PBS^−/−^, temperature equilibrate the stored Matrigel hESC-Qualified Matrix-coated flasks at 37°C for at least 15 min before use.
3. Aliquot Matrigel Growth Factor Reduced (GFR) Matrix.
  ***Note:*** Matrigel GFR Matrix has undergone the removal of growth factors during production, resulting in a more consistent composition. This enhances the stability and controllability of pancreas cell differentiation protocols.
  a. Thaw Matrigel GFR Matrix (10 mL/vial) on ice at 4°C for at least 12 h.
  b. Pre-chill 1.5 mL EP tubes and 1 mL tips at −20°C for at least 1 h.
  c. In the cell culture hood, aliquot 300 μL Matrigel GFR Matrix with pre-chilled tips into the EP tubes based on the dilution factor.
  d. Immediately store the aliquoted Matrigel GFR Matrix at −80°C.
    ***Note:*** The use of μ-Dish 35 mm ibiTreat dishes in our differentiation system enables real-time live-cell imaging throughout the differentiation process. 300 μL Matrigel GFR Matrix can seed 3 μ-Dish 35 mm ibiTreat dishes.
4. Reconstitute and prepare stocks of growth factors and small molecules (Table 1).
  a. In the cell culture hood, prepare sterile 0.1% (w/v) bovine serum albumin (BSA) solution with PBS^−/−^.
  b. In the cell culture hood, resuspend Activin A and Keratinocyte Growth Factor (KGF) powder with 0.1% BSA (Table 1).
    i. Activin A stock: 10 μg/mL, 1 mL per EP tube.
    ii. KGF stock: 100 μg/mL, 100 μL per EP tube.
      ***Note:*** Activin A and KGF stocks can be stored at −80°C for up to 1 year. Avoid more than three freeze-thaw cycles.
  c. In the cell culture hood, prepare 250 mM ascorbic acid stock by dilution in H_2_O and filter by 0.22 μm filter unit (Table 1). Aliquot ascorbic acid stock as 1 mL per EP tube.
    ***Note:*** The ascorbic acid stock can be stored at 4°C for up to 1 week or −20°C for up to 6 months.
  d. In the cell culture hood, prepare small molecule chemical compound stocks by dilution in DMSO (Table 1).
    i. Y27632 stock: 10 mM, 200 μL per EP tube. Y27632 is a ROCK1/2 inhibitor.
    ii. CHIR99021 stock: 3 mM, 50 μL per EP tube. CHIR99021 is a GSK3 inhibitor.
    iii. LDN-193189 stock: 1 mM, 20 μL per EP tube. LDN-193189 is a BMP signaling inhibitor.
    iv. SANT-1 stock: 2.5 mM, 20 μL per EP tube. SANT-1 is a Hedgehog inhibitor.
    v. Retinoic acid (RA) stock: 10 mM, 20 μL per EP tube. RA is the Vitamin A metabolite and RAR nuclear receptor agonist.
    vi. α-Amyloid precursor protein modulator stock: 0.2 mM, 100 μL per EP tube. α-Amyloid precursor protein modulator is a PKCα activator.
    vii. ALK5iII stock: 10 mM, 100 μL per EP tube. ALK5iII is a TGF-beta receptor I/activin-like kinase 5 (TGF-β-RI/ALK5) inhibitor.
    viii. γ-secretase inhibitor stock: 0.1 mM, 100 μL per EP tube. γ-secretase inhibitor is a Notch inhibitor.
      ***Note:*** These small molecule chemical compound stocks can be stored at −20°C for up to 1 year. Avoid more than three freeze-thaw cycles.
  e. In the cell culture hood, prepare 10 mg/mL Heparin stock by dilution in H_2_O and filter by 0.22 μm filter unit (Table 1). Aliquot Heparin stock into 15 mL tubes.
    ***Note:*** The Heparin stock can be stored at 4°C fridge for up to 1 month or −20°C freezer for up to 1 year.
  f. In the cell culture hood, prepare 50 mM ZnSO_4_ by H_2_O and filter by 0.22 μm filter unit (Table 1). Aliquot ZnSO_4_ stock into 15 mL tubes.
    ***Note:*** The ZnSO_4_ can be stored at 4°C for up to 1 year.
  g. In the cell culture hood, prepare 1 mM Triiodothyronine (T3) by MCDB131 medium and filter by 0.22 μm filter unit (Table 1). Aliquot T3 stock as 1 mL per EP tube.
    ***Note:*** The T3 can be stored at 4°C for up to 2 weeks or 20°C freezer for up to 1 year.

**Table 1:** Preparation of growth factors, small molecules, and BSA Fraction V for differentiation

Reagent	Stock concentration	Diluent
Activin A	10 μg/mL	0.1% BSA
KGF	100 μg/mL	0.1% BSA
Ascorbic acid	250 mM	H_2_O
Heparin	10 mg/mL	H_2_O
ZnSO_4_	50 mM	H_2_O
CHIR99021	3 mM	DMSO
LDN-193189	1 mM	DMSO
SANT-1	2.5 mM	DMSO
Retinoic acid	10 mM	DMSO
α-Amyloid precursor protein modulator	0.2 mM	DMSO
ALK5iII	10 mM	DMSO
γ-secretase inhibitor	0.1 mM	DMSO
Y27632	10 mM	DMSO
BSA Fraction V	10% (w/v)	MCDB131
Triiodothyronine (T3)	1 mM	MCDB131

Activin A and KGF stored at −80°C for up to 1 year, Ascorbic acid stored at 4°C for up to 1 week or −20°C for up to 6 months, Heparin stored at 4°C for up to 1 week or −20°C for up to 1 year, ZnSO_4_ stored at 4°C for up to 1 year, T3 and BSA Fraction V stored at 4°C for up to 2 weeks or −20°C for up to 1 year, and the rest small molecules stored at −20°C for up to 1 year.
5. Prepare 10% (w/v) BSA Fraction V stock.
  a. Resuspend 50 g BSA Fraction V powder in 500 mL MCDB131 medium and store it in the 4°C fridge for at least 12 h to allow BSA Fraction V to dissolve in the medium completely (Table 1).
  b. In the cell culture hood, filter the BSA solution by 0.22 μm filter unit to generate a 10% (w/v) BSA Fraction V stock.

***Note:*** The BSA Fraction V solution can be stored at 4°C for up to 2 weeks or −20°C freezer for up to 1 year.

### Culture of hPSCs

**Timing: 2.5 h**

At least 8 days before differentiation.

6. hPSCs thawing.
  a. Prepare 16 mL of mTESR1 plus 10 μM Y27632 medium in 50 mL Falcon tube in advance.
  b. Quickly thaw frozen cryovials containing 1 × 10^6^ hPSCs by rubbing the vials between hands.
  c. Transfer the cells into a 15 mL Falcon tube with 10 mL mTESR1 plus 10 μL Y27632 medium.
  d. Centrifuge the cells at 300 *g* for 3 min.
  e. Remove the supernatant and replace it with 5 mL mTESR1 plus 10 μL Y27632 medium by pipettes.
  f. Seed the cells to the Matrigel hESC-Qualified Matrix-coated T25 flask by pipettes.
  g. Culture the hPSCs at 37°C in an incubator with 5% CO_2_ and change the medium daily.

**Figure 1:**
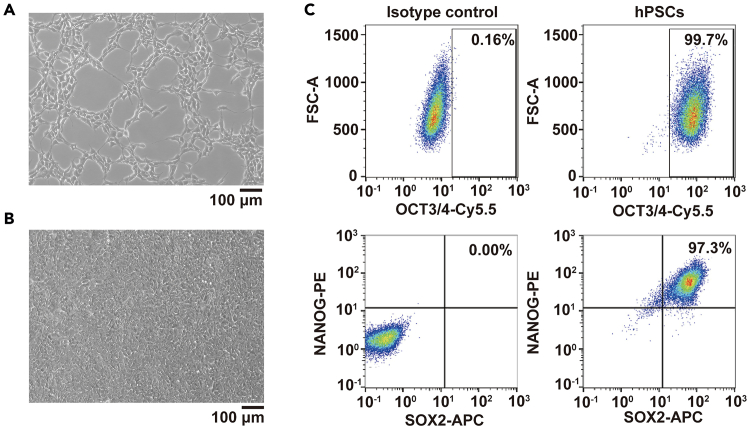
hPSC Morphology and Pluripotency (A) Morphology of hPSCs after 1 day thawing. Scale bar, 100 μm. (B) Morphology of hPSCs that are ready to be passaged (90% confluency). Scale bar, 100 μm. (C) Flow cytometry analysis of hPSCs stained with OCT3/4, NANOG, or SOX2 antibodies.

***Note:*** 1 day after thawing, the cells reach approximately 20% confluency (Figure 1A). By Day 3, the cells reach approximately 90% confluency (Figure 1B), making them ready for passaging.

7. hPSCs passaging.
  a. Prepare the Matrigel hESC-Qualified Matrix-coated T75 flask in advance.
  b. Prepare the proper volume of mTESR1 plus 10 μM Y27632 medium in 50 mL falcon tube in advance.
  c. For the flask with hPSCs scheduled for passaging, remove the medium carefully and wash the cells with PBS^−/−^ (2 mL per T25 flask; 6 mL per T75 flask) by pipettes.
  d. Remove the PBS^−/−^ and add TrypLE to T25 flask (2 mL per flask) or T75 flask (6 mL per flask) by pipettes to dissociate the cells into single cells.
  e. Incubate at 37°C for 5 min.
  f. To stop dissociation, add 2 mL of mTESR1 plus 10 μM Y27632 medium for a T25 flask or 6 mL for a T75 flask.
  g. Gently pipette up and down to dissociate the cells into a single-cell suspension.
    ***Note:*** Minimize pipetting to prevent bubble formation and maintain cell viability.
  h. Centrifuge the cells at 300 *g* for 3 min.
  i. Remove the supernatant and replace it with 5 mL of fresh mTESR1 plus Y27632 medium by pipettes.
  j. Gently mix the cells.
  k. Transfer 100 μL cell suspension by 200 μL tips to an EP tube and count the cell number by NucleoCounter cell counters.
    ***Alternatives:*** Other cell counting methods (e.g., Hemocytometer count) are also suitable for this step.
  l. Transfer 3 × 10^6^ dissociated hPSCs by 1 mL tips to a prepared Matrigel hESC-Qualified Matrix-coated T75 flask.
    ***Note:*** Cells are seeded at a density of 4 × 10^4^ cells/cm^2^ and reach 90% confluency within 3 days.
  m. Maintain the hPSCs at 37°C in an incubator with 5% CO_2_, changing the mTESR1 medium daily.
  n. Passage the hPSCs every 3 days.
    ***Note:*** Prior to initiating the differentiation process, carefully examine the hPSCs to ensure they exhibit compact morphology, uniform colony size, approximately 90% confluence, and more than 95% expression of pluripotency markers (OCT3/4, SOX2, and NANOG) (Figures 1B and 1C).

## Key resources table

**Table:** 

REAGENT or RESOURCE	SOURCE	IDENTIFIER
**Antibodies**		

EZRIN (1:500)	Abcam	#ab4069 RRID: AB_304261
Donkey anti-mouse Alexa Fluor 647 (1:500)	Thermo Fisher Scientific	#A-31571 RRID: AB_162542
Alexa Fluor 647 anti-insulin (1:40)	BD Pharmingen	#565689 RRID: AB_2739331
PE anti-glucagon (1:40)	BD Pharmingen	#565860 RRID: AB_2739382
Alexa Fluor 647 anti-NKX6.1 (1:20)	BD Pharmingen	#565891 RRID: AB_2739385
PE anti-PDX1 (1:20)	BD Pharmingen	#562161 RRID: AB_10893589
Alexa Fluor 647 anti-SOX17 (1:20)	BD Pharmingen	#562594 RRID: AB_2737670
PE anti-FOXA2 (1:20)	BD Pharmingen	# 561589 RRID: AB_10716057

**Chemicals, peptides, and recombinant proteins**		

mTESR1	STEMCELL Technologies	#85850
MCDB131	Gibco	#10372019
Corning Matrigel hESC-qualified matrix, 5 mL	Corning	#354277
Matrigel growth factor reduced (GFR) matrix, 10 mL	Corning	#CLS356231
DPBS without calcium and magnesium	Gibco	#14190-144
TrypLE Express Enzyme (1X)	Gibco	#12604013
Cell recovery solution	Corning	#CLS354253
CHIR99021	Axon Medchem	#1386
Activin A	PeproTech	#120-14
KGF	PeproTech	#100-19
Ascorbic acid	Sigma-Aldrich	#A4403
LDN-193189	Tebu Tech	#04-0074-10
SANT1	Sigma-Aldrich	#S4572
Retinoic acid	Sigma-Aldrich	#R2625
α-Amyloid precursor protein modulator	Calbiochem	#565740
ALK5iII	Santa Cruz	#sc-221234A
γ-secretase inhibitor	Merck Millipore	#565789
Heparin	Sigma-Aldrich	#H3149-100KU
Triiodothyronine (T3)	Selleck	#S5726
Bovine serum albumin (BSA) Fraction V	Roche	#10775835001
Bovine serum albumin (BSA)	Sigma-Aldrich	#B4287
Y-27632	Merck Millipore	#688000
45% glucose solution in water	Sigma-Aldrich	#G8769
7.5% sodium bicarbonate solution	Thermo Fisher Scientific	#25080094
GlutaMAX supplement	Gibco	#35050061
ZnSO_4_	Sigma-Aldrich	#Z2876
16% formaldehyde (w/v), methanol-free	Thermo Fisher Scientific	#28906
Cytofix Fixation buffer	BD Pharmingen	#554655
Perm/Wash buffer	BD Pharmingen	#554723
Triton X-100	Sigma-Aldrich	#T9284
Normal donkey serum	Jackson ImmunoResearch	#017-000-121
Fetal bovine serum	Gibco	#A5670701

**Critical commercial assays**		

BD Stemflow Human Pluripotent Stem Cell Transcription Factor Analysis Kit	BD Pharmingen	#560589
LIVE/DEAD Fixable Violet Dead Cell Stain Kit	Thermo Fisher Scientific	#L34964

**Experimental models: Cell lines**		

SA121	Takara bio	Y00020 RRID: CVCL_B296

**Software and algorithms**		

Adobe Illustrator 2023	Adobe	https://www.adobe.com
Fiji 2.0/ImageJ	NIH Image	http://imagej.nih.gov/ij
FlowJo 10	BD	https://www.flowjo.com
ZEN (blue edition)	Zeiss	https://www.micro-shop.zeiss.com

**Other**		

Cell culture incubator	Thermo Scientific	BBD 6220 CO_2_ incubator
Cell culture hood	Thermo Scientific	HERA SAFE KS
Centrifuge (cell culture)	Eppendorf	5702R
Centrifuge (flow cytometry)	Eppendorf	5430R
Centrifuge (flow cytometry)	Eppendorf	5804R
Shaker	neoLabLine	DOS-10L
Confocal microscopes	Zeiss	LSM780
Flow cytometer	Miltenyi	MACSQuant analyzer 16

## Step-by-step method details

### Establishing the hPSC-based Matrigel-overlay organoid system

**Timing: approximately 19 h**

This section provides step-by-step protocols for seeding hPSCs in the Matrigel-overlay organoid system.

1. Check that the hPSCs have reached 90% confluency and are ready for differentiation.
2. Pre-chill μ-Dish 35 mm ibiTreat dish at −20°C for at least 1 h.
3. Prepare the proper volume of mTESR1 plus 10 μM Y27632 medium in 50 mL Falcon tube in advance.
4. Remove the culture medium carefully from the cultured hPSC T75 flask and wash the cells with 10 mL PBS^−/−^ by pipettes.
5. Add 6 mL of the TrypLE per T75 flask by pipettes.
6. Incubate at 37°C for 5 min.
7. To stop dissociation, add 6 mL of mTESR1 plus 10 μM Y27632 medium by pipettes for a T75 flask.
8. Gently pipette up and down to dissociate the cells into a single-cell suspension.

***Note:*** Minimize pipetting to prevent bubble formation and maintain cell viability.

9. Centrifuge the cells at 300 *g* for 3 min.
10. Remove the supernatant and replace it with 5 mL of fresh mTESR1 plus Y27632 medium by pipettes.
11. Gently pipette up and down to mix the cells.
12. Transfer 100 μL cell suspension by 200 μL tips to an EP tube and count the cell number by NucleoCounter cell counters.

***Alternatives:*** Other cell counting methods (e.g., Hemocytometer count) are also suitable for this step.

13. Adjust cell concentration to 2.3–2.6 × 10^6^/mL with mTESR1 plus Y27632 medium.

***Note:*** The growth area of μ-Dish 35 mm ibiTreat dish is 3.5 cm^2^. The cell seeding density is 130,000-150,000 cells/cm^2^.

14. Thaw the aliquoted Matrigel GFR Matrix at 4°C for 1 h.
15. On ice, mix 600 μL cell suspension with 300 μL Matrigel GFR Matrix and gently pipette up and down by 1 mL tips to mix the cells with the Matrix.
16. Add the mixed solution into the inner growth area of μ-Dish 35 mm ibiTreat dish.

***Note:*** Make sure the mixed solution covers the whole inner growth area of μ-Dish 35 mm ibiTreat dish.

**CRITICAL:** The cell and Matrigel GFR Matrix mixture can become solid within 5 min at 20°C. This step needs to be as quick as possible. If preparing more than 3 μ-Dish 35 mm ibiTreat dishes, process them in batches of three at a time.

17. Carefully place the μ-Dish 35 mm ibiTreat dish at 4°C for 20 min to allow the cells to settle before matrix solidification.
18. Transfer the μ-Dish 35 mm ibiTreat dish from 4°C to the 37°C incubator with 5% CO_2_ and incubate for 16 h.
19. Add 3 mL mTESR1 medium to the μ-Dish 35 mm ibiTreat dish and incubator for 24 h. The cells will be ready for differentiation after the incubation.

***Note:*** The time of differentiation is recorded as Day 1.

### Differentiation of hPSCs into pancreatic endocrine cells

**Timing: 20 days**

This section provides step-by-step protocols for pancreas endocrine cell differentiation in the Matrigel-overlay organoid system. The whole procedure involves 3 different basal medium (Tables 1, 2, 3, and 4) and 6 stages (Tables 5, 6, 7, 8, 9, and 10).

***Note:*** The cells are cultured at 37°C in a 5% CO_2_ incubator for the entire differentiation process.

20. Definitive endoderm induction (3 days) (Tables 2 and 5).
  a. Day 1 of the differentiation.
    i. Carefully remove the mTESR1 medium and wash the dish with 3 mL PBS^−/−^.
    ii. Carefully remove the PBS^−/−^ and add 3 mL definitive endoderm stage 1A (Stage 1A) medium.
    iii. Allow the cells to incubate for 1 day.
  b. Day 2 of the differentiation.
    i. Carefully remove the Stage 1A medium and add 3 mL definitive endoderm stage 1B (Stage 1B) medium per dish.
    ii. Allow the cells to incubate for 1 day.
  c. Day 3 of the differentiation.
    i. Carefully remove the Stage 1B medium and add 3 mL definitive endoderm stage 1C (Stage 1C) medium per dish.
    ii. Allow the cells to incubate for 1 day.

**Table 2:** Differentiation basal medium 1 (500 mL)

Reagent	Final concentration	Amount
MCDB131	NA	453 mL
10% BSA Fraction V	0.5%	25 mL
7.5% sodium bicarbonate	0.15%	10 mL
Glutamax (100 ×)	1 ×	5 mL
45% D-Glucose	0.18%	2 mL
Antibiotic-antifungal solution (100 ×)	1 ×	5 mL

Store at 4°C for up to 1 week.

**Table 3:** Differentiation basal medium 2 (500 mL)

Reagent	Final concentration	Amount
MCDB131	NA	373.8 mL
10% BSA Fraction V	2%	100 mL
7.5% sodium bicarbonate	0.25%	16.7 mL
Glutamax (100 ×)	1 ×	5 mL
45% D-Glucose	0.18%	2 mL
Insulin-Transferrin-Selenium-Ethanolamine (ITS -X) (100 ×)	0.5 ×	2.5 mL

Store at 4°C for up to 1 week.

**Table 4:** Differentiation basal medium 3 (500 mL)

Reagent	Final concentration	Amount
MCDB131	NA	378.5 mL
10% BSA Fraction V	2%	100 mL
7.5% sodium bicarbonate	0.15%	10 mL
Glutamax (100 ×)	1 ×	5 mL
45% D-Glucose	0.36%	4 mL
Insulin-Transferrin-Selenium-Ethanolamine (ITS -X) (100 ×)	0.5 ×	2.5 mL

Store at 4°C for up to 1 week.

**Table 5:** Definitive endoderm induction, Stage 1 medium (100 mL)

Reagent	Final concentration	Amount
Differentiation basal medium 1	N.A.	99 mL
Activin A	100 ng/mL	1 mL
CHIR99021	For Stage 1A: 3 μM	100 μL
	For Stage 1B: 0.3 μM	10 μL
	For Stage 1C: 0 μM	None

Store at 4°C for up to 3 days.
21. Primitive gut tube induction (2 days) (Tables 2 and 6).
  a. Day 4 of the differentiation.
    i. Carefully remove the Stage 1C medium and wash the dish with 3 mL PBS^−/−^.
    ii. Carefully remove the PBS^−/−^ and add 3 mL primitive gut tube stage 2 (Stage 2) medium per dish.
    iii. Allow the cells to incubate for 1 day.
  b. Day 5 of the differentiation.
    i. Carefully remove the Stage 2 medium and add fresh 3 mL Stage 2 medium per dish.
    ii. Allow the cells to incubate for 1 day.

**Table 6:** Primitive gut tube induction, Stage 2 medium (100 mL)

Reagent	Final concentration	Amount
Differentiation basal medium 1	NA	100 mL
KGF	50 ng/mL	50 μL
Ascorbic acid	250 μM	100 μL

Store at 4°C for up to 3 days.
22. Posterior foregut induction (2 days) (Tables 3 and 7).
  a. Day 6 of the differentiation.
    i. Carefully remove the Stage 2 medium and add 3 mL posterior foregut Stage 3 (Stage 3) medium per dish.
    ii. Allow the cells to incubate for 1 day.
  b. Repeat one more time for 22a.

**Table 7:** Posterior foregut induction, Stage 3 medium (100 mL)

Reagent	Final concentration	Amount
Differentiation basal medium 2	NA	100 mL
KGF	50 ng/mL	50 μL
Ascorbic acid	250 μM	100 μL
Retinoic acid	1 μM	10 μL
LDN-193189	0.1 μM	10 μL
SANT-1	0.25 μM	10 μL
α-Amyloid precursor protein modulator	0.2 μM	100 μL

Store at 4°C for up to 3 days.
23. Bi-potent pancreatic progenitor induction (3 days) (Tables 3 and 8).
  a. Day 8 of the differentiation.
    i. Carefully remove the Stage 3 medium and add 3 mL bi-potent pancreatic progenitor stage 4 (Stage 4) medium per dish.
    ii. Allow the cells to incubate for 1 day.
  b. Repeat twice for 23a.

**Table 8:** Bi-potent pancreatic progenitor induction, Stage 4 medium (100 mL)

Reagent	Final concentration	Amount
Differentiation basal medium 2	NA	100 mL
KGF	2 ng/mL	2 μL
Ascorbic acid	250 μM	100 μL
Retinoic acid	0.1 μM	1 μL
LDN-193189	0.2 μM	20 μL
SANT-1	0.25 μM	10 μL
α-Amyloid precursor protein modulator	0.1 μM	50 μL

Store at 4°C for up to 3 days.

**Table 9:** Pancreatic endocrine progenitor induction, Stage 5 medium (100 mL)

Reagent	Final concentration	Amount
Differentiation basal medium 3	NA	100 mL
Retinoic acid	0.05 μM	0.5 μL
SANT-1	0.25 μM	10 μL
LDN-193189	0.1 μM	10 μL
ALK5iII	10 μM	100 μL
T3	1 μM	100 μL
Heparin	10 μg/mL	100 μL
ZnSO_4_	10 μM	20 μL

Store at 4°C for up to 3 days.
24. Pancreatic endocrine progenitor induction (3 days) (Tables 4 and 9).
  a. Day 11 of the differentiation.
    i. Carefully remove the Stage 4 medium and wash the dish with 3 mL PBS^−/−^.
    ii. Carefully remove the PBS^−/−^ and add 3 mL pancreatic endocrine progenitor stage 5 (Stage 5) medium per dish.
    iii. Allow the cells to incubate for 1 day.
  b. Day 12 of the differentiation.
    i. Carefully remove the Stage 5 medium and add fresh 3 mL Stage 5 medium per dish.
    ii. Allow the cells to incubate for 1 day.
  c. Repeat one more time for 24b.
25. Pancreatic endocrine cell induction (7 days) (Tables 4 and 10).
  a. Day 14 of the differentiation.
    i. Carefully remove the Stage 5 medium and wash the dish with 3 mL PBS^−/−^.
    ii. Carefully remove the PBS^−/−^ and add 3 mL pancreatic endocrine cell stage 6 (Stage 6) medium per dish.
    iii. Allow the cells to incubate for 1 day.
  b. Day 15 of the differentiation.
    i. Carefully remove the Stage 6 medium and add fresh 3 mL Stage 6 medium per dish.
    ii. Allow the cells to incubate for 1 day.
  c. Repeat 5 times for 25b.

**Table 10:** Pancreatic endocrine cell induction, Stage 6 medium (100 mL)

Reagent	Final concentration	Amount
Differentiation basal medium 3	NA	100 mL
LDN-193189	0.1 μM	10 μL
ALK5iII	10 μM	100 μL
γ-secretase inhibitor	0.1 μM	100 μL
T3	1 μM	100 μL
Heparin	10 μg/mL	100 μL
ZnSO_4_	10 μM	20 μL

Store at 4°C for up to 3 days.

### Characterization of differentiation efficiency and epithelial features in the differentiation

**Timing: 33 h**

This section provides step-by-step protocols for characterizing definitive endoderm, bi-potent pancreatic progenitor, and endocrine cells by flow cytometry and characterizing epithelial features at Day 13 of differentiation by immunofluorescence analysis.

#### Flow cytometry analysis for characterization of definitive endoderm, bi-potent pancreatic progenitor, and endocrine cells

**Timing: 3 h for each time point**

26. Preparation.
  a. Dilute 10× Perm/Wash buffer to 1× using PBS^−/−^.
    ***Note:*** 1× Perm/Wash buffer can be stored in the 4°C fridge for at least 1 month.
  b. Prepare the flow cytometry blocking solution by mixing 5% fetal bovine serum (FBS), 0.1% BSA, and 0.1% Triton X-100 in PBS^−/−^ (Table 11).
    ***Note:*** Flow cytometry blocking solution can be stored at 4°C fridge and used within 1 month.

**Table 11:** Flow cytometry blocking solution (100 mL)

Reagent	Final concentration	Amount
Fetal bovine serum (FBS)	5%	5 mL
BSA	0.1%	100 mg
Triton X-100	0.1%	100 μL
PBS^−/−^	NA	Make up to 100 mL

Store at 4°C for up to 1 month.
  c. Add 50 μL DMSO to one vial of Live/Dead dye tube to prepare a 1,000× Live/Dead stock solution.
    ***Note:*** 1,000× Live/Dead stock can be stored at −20°C for up to 3 months.
27. Characterization.
  a. Retrieve cells from Day 3, Day 10, or Day 20 of differentiation using Cell Recovery Solution at 4°C for 20 min.
  b. Dissociate the retrieved cells with TrypLE at 37°C for 10 min.
  c. Stop the dissociation with PBS^−/−^, filter the cells using a 50 μm strainer, and adjust the cell concentration to 1 × 10^6^ cells/mL.
    ***Note:*** Use 2 × 10^5^ cells per staining.
  d. Centrifuge at 500 *g* for 3 min and aspirate the supernatant.
    ***Note:*** The centrifugation speed was increased from 300 *g* to 500 *g* to ensure the collection of all differentiated cells.
  e. Add 1 mL 1× Live/Dead dye diluted in PBS^−/−^ to 2 × 10^5^ cells to distinguish live and dead cells.
  f. Incubate on ice for 30 min.
  g. Centrifuge at 500 *g* for 3 min and aspirate the supernatant.
  h. Add fixation buffer to the cells and incubate on ice for 10 min.
  i. Centrifuge at 500 *g* for 3 min and aspirate the supernatant.
  j. Add 1X Perm/Wash buffer to the cells and incubate on ice for 10 min.
  k. Centrifuge at 500 *g* for 3 min and aspirate the supernatant.
  l. Resuspend the cells in 200 μL of flow cytometry blocking buffer.
  m. Add the appropriate antibodies (Table 12)

**Table 12:** Antibodies used for flow cytometry

Day of differentiation	PE antibody (final concentration)	Alexa Fluor 647 antibody (final concentration)
Day 3, definitive endoderm cells	FOXA2 (1:20)	SOX17 (1:20)
Day 10, bi-potent progenitors	PDX1 (1:20)	NKX6.1 (1:20)
Day 20, endocrine cells	GCG (1:40)	INS (1:40)
  n. Incubate at 20°C in the dark for 45-60 min.
  o. Run flow cytometry analysis within 1 h.
    ***Note:*** Analyzed at least 20,000 single, live cells per sample. The live cells should be more than 85% (Figure 2).

**Figure 2:**
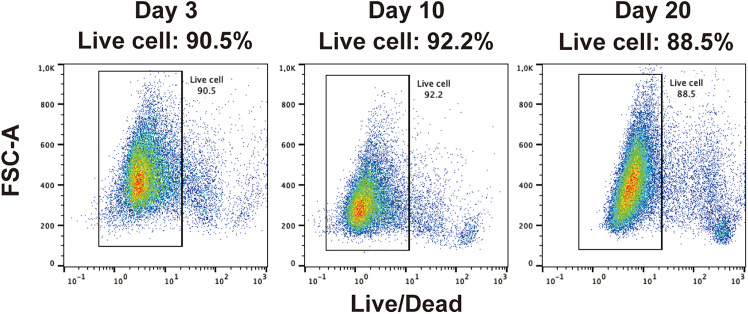
Cell viability analysis by flow cytometry Flow cytometry analysis of differentiated cells on Day 3, Day 10, and Day 20 stained with Live/Dead dye.

#### Immunofluorescence analysis for characterization of epithelial features at day 13 of differentiation

**Timing: 24 h**

28. Preparation.
  a. Prepare 0.3% Triton X-100 solution using PBS^−/−^.
    ***Note:*** Triton X-100 requires at least 1 h to dissolve fully on a shaker with 120 rpm at 20°C. 0.3% Triton X-100 solution can be stored at 20°C for at least 1 year.
  b. Prepare 5% normal donkey serum (NDS) + 0.1% Triton X-100 immunofluorescence blocking solution using PBS^−/−^ (Table 13).
    ***Note:*** Immunofluorescence blocking solution can be stored at 4°C and used within 1 month.

**Table 13:** Immunofluorescence blocking solution (10 mL)

Reagent	Final concentration	Amount
Normal donkey serum (NDS)	5%	0.5 mL
Triton X-100	0.1%	10 μL
PBS^−/−^	NA	Make up to 10 mL

Store at 4°C for up to 1 month.
  c. Prepare 4% formaldehyde by dilution of 16% formaldehyde with PBS^−/−^.
    ***Note:*** 4% formaldehyde can be stored at 4°C and used within 2 weeks.
29. Characterization.
  a. Wash cells with PBS^−/−^ and fix them with 4% formaldehyde at 20°C for 20 min.
  b. Wash cells three times with PBS^−/−^.
  c. Permeabilize with 0.3% Triton X-100 at room 20°C for 20 min.
  d. Wash cells once with PBS^−/−^.
  e. Block with immunofluorescence blocking solution at 20°C for 1 h.
  f. Aspirate the blocking solution and incubate the cells with the primary antibody (EZRIN, 1:250) at 4°C for 16 h.
  g. Wash cells three times with PBS^−/−^.
  h. Incubate the cells with the secondary antibody (Donkey anti-mouse Alexa Fluor 647, 1:500) at 20°C in the dark for 2 h.
  i. Wash cells three times with PBS^−/−^.
  j. Proceed with immunofluorescence analysis of cells kept with PBS^−/−^ in the μ-Dish 35 mm ibiTreat dishes directly.

## Expected outcomes

**Figure 3:**
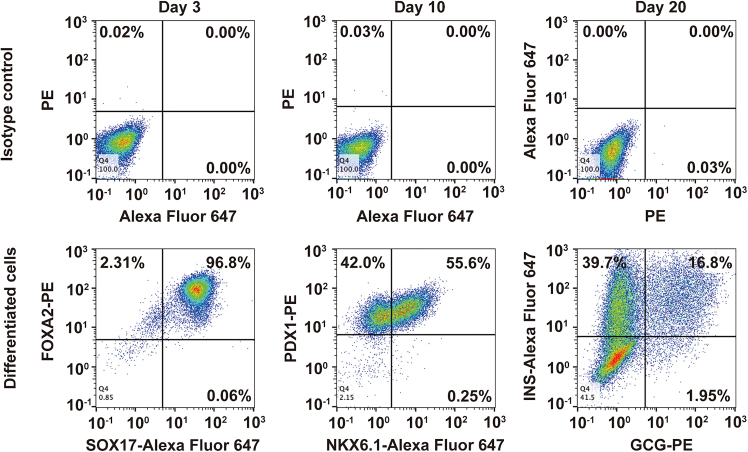
Characterization of differentiation efficiency by flow cytometry Flow cytometry analysis of differentiated cells on Day 3, Day 10, and Day 20 stained with FOXA2 and SOX17, or PDX1 and NKX6.1, or INS and GCG antibodies.

**Figure 4:**
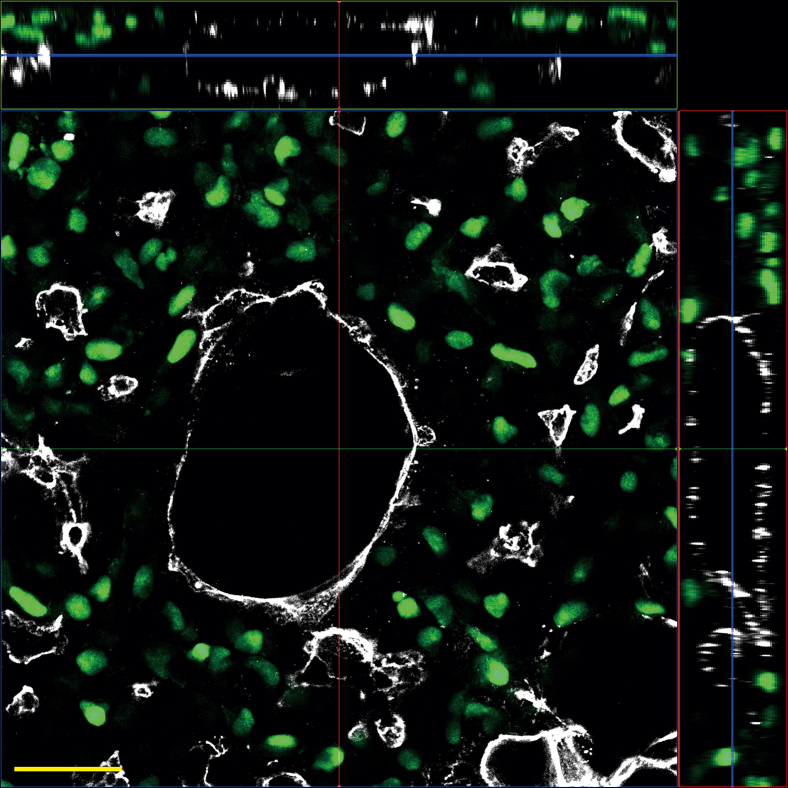
Characterization of epithelial features at Day 13 of differentiation Immunofluorescence analysis of differentiated cells on Day 13 stained with EZRIN. White, EZRIN; Green, NEUROG3-EGFP. Scale bar, 50 μm.

This protocol provides a robust approach to recapitulate the dynamic changes of the epithelial luminal structures during pancreas development *in vitro* using hPSCs. It facilitates the effective production of pancreatic endocrine cells through a meticulously refined, stepwise differentiation protocol. By the end of Day 3, more than 90% of cells express key definitive endoderm markers, such as FOXA2 and SOX17 (Figure 3). By the end of Day 10, over 50% of cells express bi-potent pancreatic progenitor markers, including PDX1 and NKX6.1 (Figure 3). By the end of Day 20, more than 55% of cells express the beta cell marker INS, and more than 17% of cells express the alpha cell marker GCG (Figure 3). The differentiation efficiency of our model system is comparable to other beta cell differentiation protocols.^7^ Also, this protocol facilitates the investigation of how microenvironmental signals regulate endocrine cell development during pancreas formation. As differentiation progresses, the cultures form epithelial luminal structures within the dish and undergo dynamic morphological changes characterized by the apical domain marker - EZRIN (Figure 4). The anticipated outcomes include the sustained formation and dynamic remodeling of epithelial luminal structures throughout differentiation (Figure 4), with a high yield of endocrine cells expressing INS and/or GCG at the end of differentiation (Figure 3). These cultures are designed to closely mimic pancreatic development in vitro, offering significant potential for both experimental research and therapeutic applications.

## Limitations

This protocol mimics pancreas specification and morphogenesis *in vitro* and efficiently differentiates hPSCs into pancreatic endocrine cells, including alpha and beta cells. However, several factors can impact overall yield, epithelial luminal structure, and consistency. First, hPSC lines with distinct genetic backgrounds may exhibit varying differentiation potentials, leading to differences in pancreatic cell differentiation efficiency. Adjusting cytokine concentrations and exposure durations based on the specific hPSC line is necessary for optimal results. Additionally, the initial seeding density of hPSCs plays a critical role in the formation of epithelial luminal structures. Therefore, optimizing the starting cell number for each hPSC line is also essential to achieve the best differentiation outcomes.

## Troubleshooting

### Problem 1

Low pluripotency of hPSCs with poor colony morphology or low pluripotent marker (OCT3/4, SOX2, NANOG) expression (related to “culture of hPSCs” section).

### Potential solution

hPSCs typically need 1-2 passages after thawing to restore stability. Moreover, for optimal differentiation outcomes, it is advisable to use hPSCs with fewer than 10 passages. Regular monitoring of pluripotency and mycoplasma contamination is essential.

### Problem 2

Inconsistent differentiation efficiency among different batches (related to “differentiation of hPSCs into pancreatic endocrine cells” section).

### Potential solution

Store cytokine aliquots at 4°C, −20°C or −80°C as specified by the manufacturer and thaw them at 4°C before use. Use only cytokines that have undergone no more than three freeze-thaw cycles, as repeated freezing and thawing can reduce efficacy.

### Problem 3

Uneven Matrigel layer or uneven cell distribution in the dish (related to “establishing the hPSC-based Matrigel-overlay organoid system” section).

### Potential solution

Complete the cell seeding process within 5 min to prevent Matrigel solidification. Prepare the required medium in advance and pre-chill the μ-Dish 35 mm ibiTreat dishes before use.

### Problem 4

Low NKX6.1 induction at the end of Stage 4 (Day 10) (related to “Pancreatic endocrine progenitor induction” section).

### Potential solution

First, verify that all cytokine preparations are correctly prepared, stored, and used as recommended (as mentioned in problem 2). Second, different cell lines may exhibit varying response times to cytokines. Adjusting cytokine concentrations and exposure durations based on the specific hPSC line is necessary for optimal results. For example, extending Stage 4 by 1-2 days may enhance NKX6.1 induction.^10^

### Problem 5

Inconsistent epithelial luminal structure among different batches (related to “establishing the hPSC-based Matrigel-overlay organoid system” section).

### Potential solution

The initial cell density is crucial for epithelial structure formation. To ensure consistency, determine the optimal hPSC seeding density for each cell line in advance. Then, uniformity in both the starting cell number and the pluripotency state of hPSCs across different batches must be maintained.

## Resource availability

### Lead contact

Further information and requests for resources and reagents should be directed to and will be fulfilled by the lead contact, Prof. Henrik Semb (henrik.semb@helmholtz-munich.de).

### Technical contact

Questions about the technical specifics of performing the protocol should be directed to and will be answered by the technical contact, Dr. Chenglei Tian (chenglei.tian@helmholtz-munich.de).

### Materials availability

This study did not generate new unique reagents.

### Data and code availability

This study did not generate or analyze any unique datasets.

## Acknowledgments

This work was supported by the European Union’s Horizon 2020 research and innovation program (ISLET, no. 874839), the Novo Nordisk Foundation Center for Stem Cell Biology (DanStem) at the University of Copenhagen (NNF grant, NNF17CC0027852), and the Helmholtz Zentrum München. U.T. received a research fellowship from the Deutsche Forschungsgemeinschaft (DFG project number 382408533).

## Author contributions

U.T. and F.H. developed the method. C.T. performed the assays, analyzed the results, and developed the figures. C.T. and H.S. wrote the manuscript.

## Declaration of interests

The authors declare no competing interests.

## References

[1] Tiemann U., Tian C., Hermann F., Proks M., Skovgaard E., Kulik I., Di Y., Sedzinski J., Semb H. (2025). Pancreatic alpha and beta cell fate choice is directed by apical-basal polarity dynamics. Dev. Cell.

[2] Lorberbaum D.S., Kishore S., Rosselot C., Sarbaugh D., Brooks E.P., Aragon E., Xuan S., Simon O., Ghosh D., Mendelsohn C. (2020). Retinoic acid signaling within pancreatic endocrine progenitors regulates mouse and human beta cell specification. Development.

[3] Mastracci T.L., Anderson K.R., Papizan J.B., Sussel L. (2013). Regulation of Neurod1 contributes to the lineage potential of Neurogenin3+ endocrine precursor cells in the pancreas. PLoS Genet..

[4] Siehler J., Blöchinger A.K., Meier M., Lickert H. (2021). Engineering islets from stem cells for advanced therapies of diabetes. Nat. Rev. Drug Discov..

[5] Larsen H.L., Grapin-Botton A. (2017). The molecular and morphogenetic basis of pancreas organogenesis. Semin. Cell Dev. Biol..

[6] Pagliuca F.W., Millman J.R., Gürtler M., Segel M., Van Dervort A., Ryu J.H., Peterson Q.P., Greiner D., Melton D.A. (2014). Generation of functional human pancreatic beta cells in vitro. Cell.

[7] Rezania A., Bruin J.E., Arora P., Rubin A., Batushansky I., Asadi A., O'Dwyer S., Quiskamp N., Mojibian M., Albrecht T. (2014). Reversal of diabetes with insulin-producing cells derived in vitro from human pluripotent stem cells. Nat. Biotechnol..

[8] Russ H.A., Parent A.V., Ringler J.J., Hennings T.G., Nair G.G., Shveygert M., Guo T., Puri S., Haataja L., Cirulli V. (2015). Controlled induction of human pancreatic progenitors produces functional beta-like cells in vitro. EMBO J..

[9] Lof-Ohlin Z.M., Nyeng P., Bechard M.E., Hess K., Bankaitis E., Greiner T.U., Ameri J., Wright C.V., Semb H. (2017). EGFR signalling controls cellular fate and pancreatic organogenesis by regulating apicobasal polarity. Nat. Cell Biol..

[10] Hermann F.M., Kjaergaard M.F., Tian C., Tiemann U., Jackson A., Olsen L.R., Kraft M., Carlsson P.O., Elfving I.M., Kettunen J.L.T. (2023). An insulin hypersecretion phenotype precedes pancreatic beta cell failure in MODY3 patient-specific cells. Cell Stem Cell.

